# Reconciling the discrepancies on the involvement of large-conductance Ca^2+^-activated K channels in glioblastoma cell migration

**DOI:** 10.3389/fncel.2015.00152

**Published:** 2015-04-20

**Authors:** Luigi Catacuzzeno, Martino Caramia, Luigi Sforna, Silvia Belia, Luca Guglielmi, Maria Cristina D’Adamo, Mauro Pessia, Fabio Franciolini

**Affiliations:** ^1^Dipartimento di Chimica, Biologia e Biotecnologie, Universita’ di PerugiaPerugia, Italy; ^2^Dipartimento di Medicina Sperimentale, Scuola di Medicina e Chirurgia, Universita’ di PerugiaPerugia, Italy

**Keywords:** glioblastoma multiforme (GBM), BK channels, migration, KCa1.1, invasion

## Abstract

Glioblastoma (GBM) is the most common and aggressive primary brain tumor, and is notable for spreading so effectively through the brain parenchyma to make complete surgical resection virtually impossible, and prospect of life dismal. Several ion channels have been involved in GBM migration and invasion, due to their critical role in supporting volume changes and Ca^2+^ influx occuring during the process. The large-conductance, Ca^2+^-activated K (BK) channels, markedly overexpressed in biopsies of patients with GBMs and in GBM cell lines, have attracted much interest and have been suggested to play a central role in cell migration and invasion as candidate channels for providing the ion efflux and consequent water extrusion that allow cell shrinkage during migration. Available experimental data on the role of BK channel in migration and invasion are not consistent though. While BK channels block typically resulted in inhibition of cell migration or in no effect, their activation would either enhance or inhibit the process. This short review reexamines the relevant available data on the topic, and presents a unifying paradigm capable of reconciling present discrepancies. According to this paradigm, BK channels would not contribute to migration under conditions where the [Ca^2+^]_*i*_ is too low for their activation. They will instead positively contribute to migration for intermediate [Ca^2+^]_*i*_, insufficient as such to activate BK channels, but capable of predisposing them to cyclic activation following oscillatory [Ca^2+^]_*i*_ increases. Finally, steadily active BK channels because of prolonged high [Ca^2+^]_*i*_ would inhibit migration as their steady activity would be unsuitable to match the cyclic cell volume changes needed for proper cell migration.

## Introduction

Glioblastoma (GBM) is the most common and aggressive primary brain tumor, and is remarkable for its high migratory and invasive potential (Holland, 2001; Nakada et al., 2007; Sforna et al., 2015). Unlike other tumors, GBMs do not disseminate through the vasculature to form metastasis in other organs. Yet they are not less lethal; they spread in fact so effectively through the brain parenchyma that at the time of diagnosis cells have already largely infiltrated the surrounding tissues making complete eradication of the tumor virtually impossible and prospect of life dismal (mean survival from diagnosis, 12–18 months). Cell migration and invasion into the healthy brain parenchyma is thus a key process we need to understand thoroughly if we want to have a chance to successfully confront this fatal tumor.

The basics of the migration process can be approximated by cells moving in a culture dish on a flat two-dimensional (2D) surface. Cell migration in 2D encounters no steric hindrance or frictional forces arising from the surrounding environment, and can be modeled as a strictly controlled cyclic sequence of events consisting of initial protrusion of the cell leading edge, its adhesion to the substrate, actin cytoskeletal rearrangements, cell contraction and detachment of adhesion sites at the rear end (Li et al., 2005). By contrast, invasion of tumor cells into brain parenchyma can be better approximated by cells moving inside a 3D gel matrix, where invading cells have to overcome forces that arise from the steric hindrance of the matrix network, provided that the pores through which the cell moves are smaller than the cell itself. Cells try to overcome these obstacles by changing shape and volume to fit through the extracellular matrix (ECM) trabecular structure, and trying to digest and deform the ECM to widen the pores for their passage (Cuddapah and Sontheimer, 2011; Vehlow and Cordes, 2013). Regardless of the type of migration, Ca^2+^ signals play a major regulatory role, as they target most of the proteins involved (Wei et al., 2012). Notably, repetitive Ca^2+^ oscillations due to the cyclic release of Ca^2+^ from the intracellular stores have been implicated in the migration of GBM cells (Bordey et al., 2000; Rondé et al., 2000; Ishiuchi et al., 2002; Lyons et al., 2007; Catacuzzeno et al., 2011). These cyclic Ca^2+^ waves induced by serum or more specific extracellular agonists are essential for the migratory process, as they promote the cell rear detachment from the substrate and the volume changes accompanying cell rear retraction (Giannone et al., 2002; Watkins and Sontheimer, 2011).

GBM cell invasion critically depends on ion channels, determinant for dynamic volume regulation and intracellular Ca^2+^ signals. As for cell volume regulation, the prevailing view considers the concerted opening of K and Cl channels with the resulting efflux of KCl and osmotic water as the critical step underlying cell shrinkage during GBM cell migration and infiltration (Catacuzzeno et al., 2014; Turner and Sontheimer, 2014). The Cl^−^ efflux involved in this process has been shown to be mainly sustained by the ClC-3 type Cl channel. As for the K^+^ contribution, two Ca^2+^-activated K (KCa) channels have the properties for playing major roles in the process—the large (BK, KCa1.1) and intermediate (IK, KCa3.1) conductance Ca^2+^-activated K channels. Not only these channels concur in controlling the cell volume; by modulating the membrane potential they affect the driving force for Ca^2+^ influx and thus exert a secondary control on cell migration through regulation of [Ca^2+^]_*i*_ signals. The relevance of IK channels to GBM cell migration is now largely established (Fioretti et al., 2006; Sciaccaluga et al., 2010; Catacuzzeno et al., 2011, 2012a; Ruggieri et al., 2012; D’Alessandro et al., 2013). The contribution of BK channels to migration is instead less clear. Available data are not always consistent with a well-defined role of this channel type in cell migration/invasion. While BK channels inhibition typically resulted in restraint of cell migration, and in some instances was ineffective, their activation would either enhance or inhibit migration. In other words, evidence supporting their participation in GBM cell migration/invasion is still uncertain, or conflicting at times. This review will focus on and examine the role of BK channels in migration and invasion of GBM.

## BK Channel Properties and Modulators

BK channels, encoded by the *KCNMA1* gene and expressed in virtually all tissues, are regulated by both [Ca^2+^]_*i*_ and membrane potential. They have the typical tetrameric structure of K channels, with four α-subunits each displaying seven transmembrane segments, with a unique S0 segment, and the charged S4 segment conferring the voltage-dependence. Ca^2+^ sensitivity comes instead from the bulky C-terminal tail that includes a negatively charged, high-affinity Ca^2+^ binding region (the Ca^2+^ bowl; Schreiber and Salkoff, 1997; Jiang et al., 2001), and the double negative charged RCK-domain (regulatory domain for K conductance). Notably, in 2002 Sontheimer’s lab identified a splice variant of the BK channel on a human glioma cell line (D54), which they named gBK (g for glioma), being highly expressed in GBM cells but not in normal tissue (Liu et al., 2002). This variant has a significantly higher sensitivity to [Ca^2+^]_*i*_ than normal BK channels, and can be activated by agents that raise the [Ca^2+^]_*i*_ to several hundred nanomolar (Ransom et al., 2002), concentrations relatively high, yet observed in the presence of extracellular Ca^2+^ agonists.

Here we will recall the principal features of the BK channel modulators that will be encountered below, namely the BK channel blockers tetraethylammonium (TEA), iberiotoxin (IbTx) and paxilline, and the BK channel openers NS1619 and phloretin. TEA is a rather unspecific K channel blocker. However, among the TEA-sensitive K channels, the BK channel results one of the most sensitive to TEA (IC_50_ below 1 mM). For this reason, concentrations of TEA of a few millimolar may be considered a pharmacological tool to selectively inhibit BK channels in GBM cells, where other TEA-sensitive K channels are not expressed. IbTx is a 37 aminoacid peptide purified from the scorpion *Buthus tamulus*, that selectively blocks the BK channel at nanomolar concentrations from the external side, by driving the channel into a closed state that can last minutes. Paxilline is a fungal alkaloid that potently inhibits BK channels in a state-dependent manner, as it binds more tightly to the closed state. Binding appears to occur at a superficial location near the external vestibule, but does not prevent access of smaller molecules to this cavity (Zhou and Lingle, 2014). Unfortunately, paxilline has also been shown to inhibit the endoplasmic reticulum Ca-ATPase (SERCA; IC_50_ of 5–50 µM depending on the specific type of the transporter; Bilmen et al., 2002) and to sensitize the TRAIL (TNF-related apoptosis-inducing ligand)-induced GBM cell death, an effect mimed by other SERCA inhibitors, but not by other BK channel inhibitors (Kang et al., 2011). These results suggest caution when drawing conclusions on the role of BK channels using paxilline as the only BK channel inhibitor. NS1619 is a potent benzimidazolone BK channel opener (EC_50_ 10–30 µM; Olesen et al., 1994). It has been recently shown in C2C12 and H9C2 cells that NS1619 releases Ca^2+^ from internal stores and prevents Ca^2+^ accumulation by the SR vesicles (Wrzosek, 2014). In addition, NS1619 effectively inhibits IK channels also expressed in GBM cells (Warth et al., 1999; our unpublished observation in GBM cells), suggesting caution when interpreting data on the functional effects of this compound.

## Literature Survey on BK Channels and GBM Cell Migration

To verify whether cell shrinkage determined by K^+^ and Cl^−^ ion efflux is a requirement for tumor cells invasion through narrow extracellular spaces in the brain, Soroceanu et al. (1999) tested the effects of a number of Cl and K channel blockers on GBM cells migration, using different migration assays. They found both IbTx and TEA (1 mM) to effectively inhibit GBM transwell migration through 8-µm pores coated with the ECM component vitronectin. TEA was also able to inhibit GBM cell invasion through both cellular aggregates of fetal rat brain and normal brain slices, but did not modify 2D GBM cell migration tested on culture dishes by time lapse video-microscopy. These results led the authors to postulate that BK channels give an important contribution only when GBM cell migration occurs through the narrow extracellular spaces that characterize the brain parenchyma, where volume and shape changes are especially needed.

Another study prompted by the observation that patients with brain tumors often have high levels of acetylcholine (ACh) in their cerebrospinal fluid showed that raising the [Ca^2+^]_*i*_ by muscarinic activation of ACh receptors activated BK channels and inhibited U373 cell migration tested with a 2D motility assay (Bordey et al., 2000). These results were confirmed few years later on another GBM cell line by Kraft et al. (2003), who also found that activation of BK channels with NS1619 and phloretin had similar inhibitory effects on 2D motility. They found however that the BK channel activator phloretin also increased the resting [Ca^2+^]_*i*_ (from 235–470 nM). Notably, the effects of phloretin and ACh on cell migration were abolished by co-application of the specific BK channel blockers paxilline and IbTx, while the same blockers had no effects on the basal cell migration (i.e., migration in the absence of BK channel activators). These findings indicate that BK channel activation by increased [Ca^2+^]_*i*_ or selective agonists results in inhibition of GBM cell migration.

Based on the notion that 2D migration assays, as used in the previous studies, do not exactly mimic the type of migration taking place when GBM cells infiltrate through the brain parenchyma *in vivo*, Sontheimer and coworkers reexamined the effects of BK channel inhibitors and activators on GBM cell migration using the transwell migration assay with 8 µm pores, a size small enough for typical GBM cells to pass through only upon significant shrinking (Weaver et al., 2006). Under these conditions they found that inhibition of BK channels with either IbTx or paxilline markedly reduced migration of GBM cells. These results, essentially in conflict with the previous studies, seemed to give credit to the notion that different migration modes are regulated in different ways by BK channels. Namely, when migration requires significant cell volume changes, such as those needed to pass through narrow pores, BK channels are important since they concur significantly to sustaining the K^+^ efflux required for cell shrinkage.

Another investigation addressed the role of BK channels on migration of GBM cells using menthol, an agonist of the transient receptor potential melastatin 8 (TRPM8) channel, to increase [Ca^2+^]_*i*_ (Wondergem and Bartley, 2009). Menthol was found to activate BK channels, and to increase the rate of DBTRG cell migration, an effect that was inhibited by paxilline and TEA. The same inhibitors were instead ineffective when tested on basal migration (i.e., in the absence of menthol). These results, obtained with a 2D migration assay (scratch wound), were at odds with the data previously reported, and seemed to rule out the interpretation that the involvement of BK channels in GBM cell migration depends on the migration type. Notably, this study also found that inhibition of BK channels not only restrained menthol-induced cell migration, but also the menthol-stimulated [Ca^2+^]_*i*_ increase, suggesting that BK channels also help raise [Ca^2+^]_*i*_, which they do by hyperpolarizing the cell and increasing the electrochemical potential for Ca^2+^ influx.

Based on previous observations showing that migration of GBM cells is stimulated by ionizing radiation (IR), Steinle et al. (2011) tested whether IR-induced GBM cell migration involved BK channels. Their study showed that IR activated BK channels and enhanced transwell migration in a paxilline-dependent manner in T98G and U87MG cells. They further observed that IR also activated the Ca^2+^/calmodulin-dependent kinase II (CaMKII), and that its inhibition by KN-93 abolished the IR-stimulated migration. Based on these data, these authors concluded that IR stimulates BK channel activity which results in activation of CaMKII leading to enhanced cell migration.

## Reconciling Data on the Involvement of BK Channels in GBM Cell Migration

**Figure 1:**
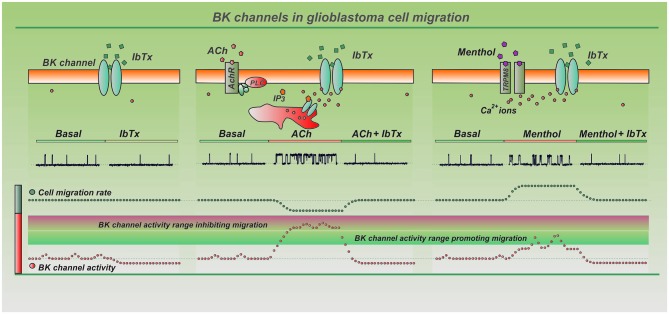
**Sketch illustrating the proposed unifying view to reconcile present data on the involvement of BK channels in glioblastoma (GBM) cell migration**. The Figure reports in separate panels the impact of three distinct experimental conditions on BK channel activity and cell migration, namely, absence of BK channel or Ca^2+^ agonists (first panel), and presence of two different Ca^2+^ agonists (ACh and menthol) that raise [Ca^2+^]_*i*_ to markedly different values (second and third panels). Each panel reports, from top down, a sketch of the major elements involved, as ion channels, membrane receptors, signaling pathways, [Ca^2+^]_*i*_, BK channel agonists and inhibitors. Lower level shows reconstructed BK single channel current traces indicating the level of channel activity under the various conditions. The bottom part illustrates the BK channel activity as qualitatively derived from BK single channel data above, and the corresponding GBM cell migration rate. *First panel* shows that BK channel antagonists are ineffective in inhibiting basal migration. This possibly indicates that under these conditions BK channels do not contribute sensibly to basal migration, consistent with the finding that at the resting [Ca^2+^]_*i*_ (100–200 nM) and membrane potential of GBM cells (30–50 mV negative) the BK channel activity is close to zero. *Second panel* shows that ACh inhibits cell migration, and the effect is reversed by IbTx. ACh has been reported to increase [Ca^2+^]_*i*_ to a level well above 750 nM, a value capable to activate the BK channels of resting cells. These data thus suggest that a high and steady activation of BK channels exerts a negative effect on migration, possibly because this high and steady K^+^ efflux does not tune with the cyclic cell volume changes needed for cell migration. In this context it is relevant to notice that menthol *(third panel)*, that activates the transient receptor potential melastatin 8 (TRPM8) channel and increases resting [Ca^2+^]_*i*_ only to ca. 300 nM, a value too low to activate BK channels of a resting cell, promotes 2D cell migration. However, if we consider the additional increments of [Ca^2+^]_*i*_ due to serum-induced [Ca^2+^]_*i*_ oscillations, BK channels would be cyclically activated during menthol exposure at the oscillatory peaks, and in this way serve nicely the cyclical K^+^ efflux needed for cell migration.

In the previous section we have reviewed the available data on the contribution of BK channels to GBM cell migration, and found a highly diversified outcome oscillating from a negative to null, to positive action. In the following, we will try to work out a paradigm capable of reconciling the presently available data on BK channel involvement in GBM cell migration into a unifying model (Figure 1). In this undertaking we consider as primary players the cell Ca^2+^ signaling and the migration mode.

*2D cell migration in the absence of* Ca^2+^* and BK channel agonists—*If we restrict the analysis of the experimental data to those obtained in 2D migration assays in the absence of Ca^2+^ or BK channels agonists, the results obtained by the various groups become essentially coherent. Monitoring single U251 cell migration by 2D time-lapse video-microscopy, Soroceanu et al. (1999) found that 1 mM TEA was ineffective on 2D cell migration. Kraft et al. (2003), using the same technique, reported that 100 nM IbTx and 2 µM paxilline were ineffective in inhibiting basal migration of 1321N1 cells. Likewise, Wondergem and Bartley (2009) reported that 10 mM TEA and 2 µM paxilline were ineffective on the wound healing 2D assay performed on DBTRG cells. All these data indicate that BK channels do not contribute to the basal 2D migration, consistent with the finding that at the resting [Ca^2+^]_*i*_ (100–200 nM; Bordey et al., 2000; Kraft et al., 2003) and membrane potential of GBM cells (−50 to −20 mV; Ransom et al., 2002; Fioretti et al., 2009), the BK channel activity is close to zero (see Ramson et al., 2002 for GBM BK channel activity at varying [Ca^2+^]_*i*_ and membrane potentials). Not even serum, present in all the migration assays referred to above, and shown to promote [Ca^2+^]_*i*_ oscillations in U87MG cells (Rondé et al., 2000; Giannone et al., 2002), was able to activate BK channels at any significant level under these experimental conditions, as expected from previous studies (Catacuzzeno et al., 2011, 2012b). The absence of a role of BK channels in basal 2D migration may thus be the result of their inactivity at the [Ca^2+^]_*i*_ and membrane potentials typical of GBM cells in a culture dish (Figure 1, left panel).

*2D cell migration in the presence of* Ca^2+^* and BK channel agonists—*Kraft et al. (2003) reported that phloretin and ACh inhibited 2D cell migration, and the effect was reverted by IbTx (100 nM) and paxilline (2 µM). They also showed that phloretin, besides directly activating the BK channel, increased the [Ca^2+^]_*i*_ to values close to 500 nM, and these two effects combined brought to BK channel activation at the resting potential of GBM cells. Bordey et al. (2000) similarly found that ACh, acting on muscarinic receptors, increased the [Ca^2+^]_*i*_ to a level well above 800 nM, a value capable to activate the BK channels of resting cells (Ransom et al., 2002). These data suggest that a marked and steady activation of BK channels, attained either by increasing the [Ca^2+^]_*i*_ or using BK channel activators, exerts a negative control on 2D migration, possibly because this constant K^+^ efflux does not tune with the cyclic cell volume changes needed during migration (Figure 1, center panel). In this context, Wondergem and Bartley (2009) reported that menthol, unlike ACh and phloretin, promotes 2D cell migration tested by wound-healing assay, an effect that was reverted by paxilline and TEA. Interestingly, menthol increased the resting [Ca^2+^]_*i*_ only to about 300 nM, a value much lower than that induced by ACh and phloretin, and too low to activate BK channels in a resting cell. However, if we consider the additional increments of [Ca^2+^]_*i*_ due to serum-induced [Ca^2+^]_*i*_ oscillations (Catacuzzeno et al., 2011), BK channels would be cyclically activated at the oscillatory peaks, and in this way allow the cyclical activity needed for cell migration (Figure 1, right panel).

*3D cell migration—*Soroceanu et al. (1999) reported that 10 nM IbTx and 1 mM TEA inhibited the 3D transwell migration of U251 GBM cells by 20% and 50%, respectively. In accordance Weaver et al. (2006) reported that 100 nM IbTx and 2 µM paxilline inhibited the 3D transwell migration by 60% and 80%, respectively. These data indicate that, in contrast to what has been observed in 2D cell migration assays, in 3D migration BK channels have a prominent role also under basal, unstimulated conditions, i.e., in the absence of Ca^2+^ or BK channel agonists. This implies that in 3D transwell migration assays, BK channels have a significant activity even in the absence of extracellular agonists, possibly due to an elevated resting [Ca^2+^]_*i*_ level. Interestingly, in the 3D transwell migration assays reported above the pore filters were routinely coated with vitronectin, an ECM component secreted by GBM cells, that has been shown to increase [Ca^2+^]_*i*_ through its interaction with plasma-membrane integrins (Schwartz, 1993; Shankar et al., 1995).

## Conclusions

Based on the above data, the involvement of BK channels in GBM cell migration, more than on the cell migration mode (2D vs. 3D), appears to primarily depend on the features of the Ca^2+^ signals (i.e., steady vs. oscillatory), and ultimately on the degree and mode (again, steady vs. oscillatory) of BK channel activation. More specifically, for [Ca^2+^]_*i*_ too low for BK channels to be activated, they will not obviously be able to contribute to cell migration. By contrast, an intermediate [Ca^2+^]_*i*_, as that induced by menthol, although insufficient to activate BK channels, is decisive as it predisposes the background for BK channels cyclic activation following oscillatory [Ca^2+^]_*i*_ increases, such as those induced by serum or other Ca^2+^ agonists. This type of cyclic BK channels activity represents an important contribution to cell migration, possibly by underlying the cyclic cell volume changes needed during migration. Finally, very high [Ca^2+^]_*i*_, such as those generated by ACh and phloretin, that keep BK channels steadily (continuously) active will disfavor cell migration because a steady K^+^ efflux is unsuitable to match the cyclic cell volume changes needed for proper cell migration.

## Conflict of Interest Statement

The authors declare that the research was conducted in the absence of any commercial or financial relationships that could be construed as a potential conflict of interest.
